# Working from home and intimate partner violence among cis-women during the COVID-19 pandemic: evidence from a global, cross-sectional study

**DOI:** 10.1186/s12889-023-15785-7

**Published:** 2023-05-26

**Authors:** Naomi Miall, Suzanna C. Francis, Heidi Stöckl, Joseph D. Tucker

**Affiliations:** 1 Faculty of Epidemiology and Population Health, London School of Hygiene and Tropical Medicine, LSHTM, Keppel Street, London, WC1E 7HT UK; 2 MRC International Statistics & Epidemiology Group, Faculty of Epidemiology and Population Health, London School of Hygiene and Tropical Medicine, LSHTM, Keppel Street, London, WC1E 7HT UK; 3 Institute of Medical Information Processing, Biometry and Epidemiology (IBE), Faculty of Medicine, LMU Munich, Marchioninistr. 15, 81377 München, Germany; 4 Department of Clinical Research, London School of Hygiene and Tropical Medicine Keppel Street, Keppel Street, London, WC1E 7HT UK; 5 Institute for Global Health and Infectious Diseases, University North Carolina, 130 Mason Farm Rd, Chapel Hill, North Carolina UK

**Keywords:** Domestic abuse, Employment, COVID-19 pandemic, Homeworking

## Abstract

**Background:**

Intimate partner violence (IPV) may have been exacerbated during the COVID-19 pandemic. This analysis aimed to determine how employment disruption during COVID-19, including working from home, was associated with IPV experience among cis-gendered women.

**Methods:**

The International Sexual Health and Reproductive health (I-SHARE) study is a cross-sectional online survey implemented in 30 countries during the pandemic. Samples used convenience, online panel, and population-representative methods. IPV was a pre-specified primary outcome, measured using questions from a validated World Health Organisation instrument. Conditional logistic regression modelling was used to quantify the associations between IPV and changes to employment during COVID-19, adjusted for confounding.

**Results:**

13,416 cis-gender women, aged 18–97, were analysed. One third were from low and middle income countries, and two thirds from high income countries. The majority were heterosexual (82.7%), educated beyond secondary-level (72.4%) and childless (62.7%). During COVID-19 33.9% women worked from home, 14.6% lost employment, and 33.1% continued to work on-site. 15.5% experienced some form of IPV. Women working from home experienced greater odds of IPV than those working on-site (adjusted OR 1.40, 95% CI 1.12–1.74, p = 0.003). This finding was robust independent of sampling strategy and country income. The association was primarily driven by an increase in psychological violence, which was more prevalent than sexual or physical violence. The association was stronger in countries with high gender inequality.

**Conclusions:**

Working from home may increase IPV risk globally. Workplaces offering working from home should collaborate with support services and research interventions to strengthen resiliency against IPV.

**Supplementary Information:**

The online version contains supplementary material available at 10.1186/s12889-023-15785-7.

## Background

One third of all women will experience physical or sexual abuse by a partner or sexual violence by a non-partner in their lifetime [1]. Intimate partner violence (IPV) is defined by the World Health Organization (WHO) as “any act or omission by a current or former intimate partner which negatively effects the well-being, physical or psychological integrity, freedom, or right to full development” of the survivor [2]. IPV is a critical public health issue causing physical injury, lingering mental health problems, and emotional trauma [3]. An understanding of the risk factors for IPV can help to inform prevention, management, and structural responses.

The relationship between a women’s employment status and IPV experience is complex. Employed women may gain economic and decision-making empowerment that can be protective against IPV, especially in contexts where women’s financial autonomy is normalised [4, 5]. However, in contexts where strict gender hierarchies are normalised, economic independence can also increase IPV risk [5], and several studies have found that women’s employment is associated with increased IPV, especially if their partner does not work or earns less [4, 6]. It is not yet clear whether the association between IPV and employment was different in the context of the COVID-19 pandemic, when both homelife and workplaces were highly disrupted. Some, but not all studies from early in the pandemic have suggested that unemployment was associated with higher IPV during this period [7, 8]. However, no studies have yet assessed the impact of working from home on IPV.

Working from home, defined as generating income at home and not including unpaid domestic labour [9], was increasingly common prior to 2020 [10]. COVID-19 accelerated this trend, with workplaces across low-, middle-, and high-income countries providing mandatory or optional home working during the pandemic [11]. In many workplaces working from home has continued into 2022. For example, in the UK one in four adults worked from home (exclusively or partially) between April and May 2022 [12]. However, there is substantial uncertainty about how working from home impacts IPV. Working from home could decrease IPV. For example, abusers may be more able to monitor their partners, and so perceive less need to use to violence for control [13]. Additionally, during the COVID-19 pandemic those attending their normal worksite may have been at greater risk of COVID-19 infection, which could be a source of relational tension [14]. However, it is more likely that working from home might increase IPV by increasing a woman’s exposure to an abusive partner [15], seeding tension in a relationship, or removing sources of resilience that employment can provide, including authority and close social contacts outside the home [16, 17].

The International Sexual Health and Reproductive Health (I-SHARE) survey is a large, global cross-sectional study conducted between 2020 and 21. Demographic and country-level correlates of IPV in this dataset have been reported elsewhere [18]. This analysis seeks to use the I-SHARE data explore the association between employment, especially working from home and IPV during COVID-19 among cis-women. We focused this analysis on cis-gender women to avoid overshadowing the experiences of non-cis gender identities by homogenising groups. IPV causes and consequences are closely entwined with gender [19], with transgender women experiencing greater risk of severe IPV [20]. The results have the potential to inform future decisions on working from home policies.

## Methods

### Study design and data sources

The study population was drawn from the I-SHARE survey, which collected information about relationships, health and employment during the pandemic. The protocol, including pre-specified outcomes, has been published elsewhere [21]. In summary, an online survey, accessed on a personal device, was distributed to 23,067 adults in 30 countries between July 2020 and February 2021. Population representative samples were recruited in two countries; six countries surveyed online panels with members selected based on gender, age, ethnicity and residence; the remaining samples were recruited by convenience methods, through email listservs, social media and local sexual and reproductive health networks (Appendix A). The survey underwent local field-testing with at least 10 participants in each country to review translation and the use of sensitive topics, local ethical review and Institutional Review Board approval in each country. Measures to protect participant safety included making all questions optional and confidential, providing information and contact details for local support organizations, and distributing the survey on personal devices to facilitate completion in private [22]. All participants were required to provide informed consent. Ethical approval for this secondary analysis was granted by the London School of Hygiene and Tropical Medicine Ethics Committee.

Stringency of restriction policies experienced was measured using data on country-level pandemic responses, obtained from the Oxford COVID-19 Government Response Tracker (OX-CGRT) [23]. This open access dataset contains a daily updated index scoring the stringency of containment policies in each country. The index summarises school, workplace and travel closures, stay-at-home rules and health information campaigns. A mean of the relevant country’s stringency index over the period (between 1 and 12 months) each participant had been in lockdown was calculated and assessed as a potential confounder. Adjustment for lockdown stringency did not change the main effect estimate so this was not included in the final adjustment model.

### Study population

Of the 23,067 participants in I-SHARE, 13,457 (58.7%) identified as cis-women. It was decided *a priori* that countries with fewer than 20 participants would be excluded to reduce bias, which meant that 41 women from four countries were excluded (Appendix A). A sensitivity analysis including these women showed almost identical results (Appendix B). Duplicates were removed in Czechia, where two sampling schemes were used.

### Measures

IPV was measured using five items from a validated WHO instrument that measure physical, psychological and sexual IPV [24]. Participants were asked to choose the frequency with which they experienced specific acts of psychological, sexual and physical violence (Appendix C), during COVID-19 social distancing measures. To define this period, a date marking the start of local restrictions was chosen by the research team in each country. A participant was defined as experiencing IPV if she answered “yes once”, or “yes multiple times” to any of the violence indicators. These items were optional, and those who did not answer all five items were classed as non-responding. Experience of physical and sexual violence, or psychological violence (encompassing emotional abuse and limits on social contacts) were explored separately as secondary outcomes.

The primary exposure was working from home during the pandemic. The effect of losing employment or working hours was also assessed. Three other types of work disruption were grouped in an “other” category, because they affected few participants and had less clear relevance to future policy. This included those who were employed but unable to work, changed job, or responded that they experienced some “other” work disruption, which could not be specified in free text. These exposures were compared to a baseline group of individuals who were working in the same job and site or were retired.

Demographic variables included as potential confounders were age, sexual orientation, ethnicity, educational attainment, marital status, motherhood, and urban location. Other variables that were tested as confounders included experiences prior to the pandemic relating to employment and participation in transactional sex, alcohol and cannabis use, household income, and prior experience of IPV. The following variables related to experiences during the pandemic were also tested: living with a partner; changes in household composition; stringency of social distancing measures experienced; and length of time spent in lockdown. The processing of these variables is described in Appendix D.

### Statistical analysis

Descriptive statistics were used to summarise the prevalence of each exposure and potential confounder. The prevalence of each form of IPV were tabulated by country.

Associations between the types of work disruption and IPV were calculated using a conditional logistic regression model, with country as a fixed effect. A parsismonious set of confounders was identified using a forward modelling strategy and minimisation of mean squared error [25], with age and prior employment status included *a priori*. This led to a final estimate adjusted for country, age, prior employment status, cannabis use before COVID-19, and previous experience of IPV in the three months before COVID-19 (Model 2). Further addition of any of the potential confounders listed above did not change the effect size compared to the crude model which excludes those with missing data on that variable.

A minimally adjusted model was fitted (Model 1), only adjusting for country as a fixed effect and excluding those with missing data on variables in Model 2 to make unbiased comparisons (N = 8362).

Missing data patterns were described by cross-tabulating outcome nonresponse with the other variables of interest. Multiple imputation was used to explore the results’ sensitivity to missing outcome data (method detailed in Appendix E). Any association between IPV and nonresponse on other modelled variables was also explored using cross-tabulations. The analysis was repeated, stratified into high-income countries (HIC) or low- and middle-income countries (LMIC), stratified based on country-level gender inequality, stratified by whether participants were living with a partner, restricted to probability-based samples, and including those with partial information on IPV as sensitivity tests. All analysis was performed using STATA/SE 16.1.

## Results

**Fig. 1:**
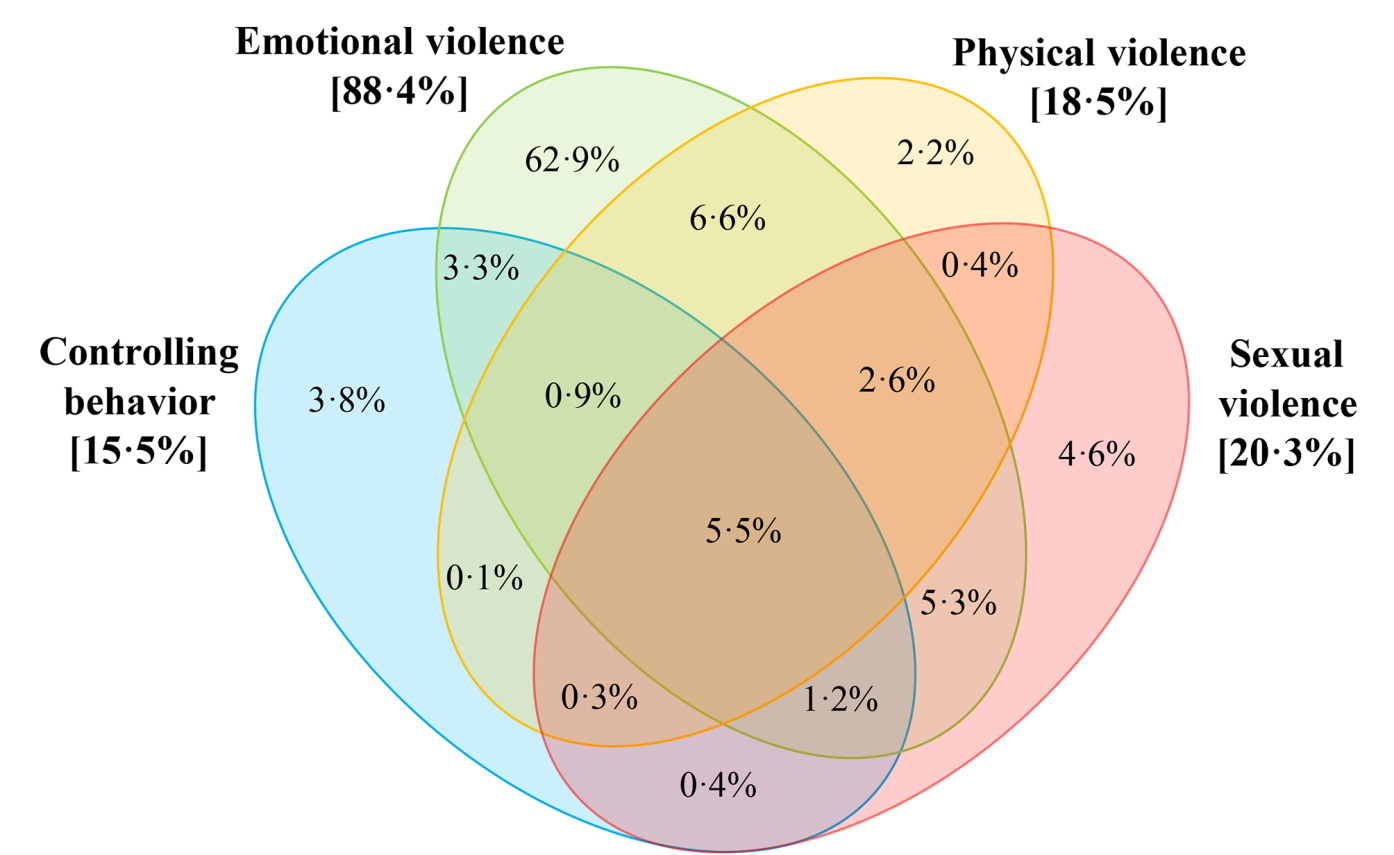
Co-occurrence of physical violence, sexual violence, emotional violence and controlling behaviour Legend: Overlap between different forms of intimate partner violence experienced during COVID-19 social distancing measures amongst 1392 cis-gendered women who experienced any IPV during this period, and for whom data was available for all four types of violence in the I-SHARE 2020-21 survey. Please note the area under the curve does not correspond to the proportion represented

Out of the 9004 participants (67.1% of the study population) who responded to questions about IPV, 15.5% had experienced any form of IPV during COVID-19 social distancing measures (Table 1). The most common was emotional abuse, experienced by 13.8%. The prevalence of sexual abuse (3.2%) was similar to physical abuse (3.0%). 2.5% of participants’ partners had attempted to restrict their social contacts (including online or telephone contact). Of those who experienced violence, 26.5% experienced multiple forms of IPV and 5.5% experienced all four forms (Fig. 1). Furthermore, of those who experienced violence during COVID-19, 80.0% experienced violence in the three months prior to COVID-19 lockdowns in their country, 15.2% did not experience violence prior to COVID-19 and 4.7% did not respond to survey items about prior violence experience (Appendix F).

**Table 1:** Prevalence of emotional, physical or sexual abuse or controlling behaviour by an intimate partner experienced during COVID-19 social distancing measures by 13,416 cis-gendered women who participated in the I-SHARE survey 2020-21 in 26 countries, of whom 9004 responded to questions on violence

World Bank Economy Classification	Country	Sample size (proportion of total sample)	Respondents to violence items (% of country sample)	Type of intimate partner violence				
				Emotional abuse ^a^ (%^f^)	Sexual abuse ^b^ (%^f^)	Physical abuse ^c^ (%^f^)	Limited contact ^d^ (%^f^)	Any violence ^e^ (%^f^)
Low Income	Uganda	108 (0.8)	70 (64.8)	20.0	12.9	5.7	2.9	27.1
	Mozambique	35 (0.3)	28 (80.0)	25.0	3.6	3.6	0.0	28.6
	Total	143 (1.1)	98 (68.5)	21.4	10.2	5.1	2.0	27.6
Lower-Middle Income	Moldova	189 (1.4)	157 (83.1)	13.1	1.3	3.1	0.0	13.4
	Nigeria	129 (1.0)	43 (33.3)	13.0	4.7	4.4	4.8	14.0
	Kenya	161 (1.2)	92 (57.1)	23.4	12.9	7.5	12.8	29.4
	Total	479 (3.6)	292 (61.0)	16.3	5.4	4.7	4.7	18.5
Upper-Middle Income	Argentina	677 (5.1)	442 (65.3)	16.9	1.1	1.1	0.9	18.1
	Botswana	278 (2.1)	151 (54.3)	14.8	5.2	6.6	1.9	19.9
	Colombia	1482 (11.1)	969 (65.4)	18.2	3.8	3.2	1.9	20.0
	Malaysia	112 (0.8)	60 (53.6)	21.3	1.6	4.8	1.6	23.3
	Mexico	1206 (9.0)	713 (59.1)	22.5	3.6	4.6	3.0	25.3
	China	153 (1.1)	131 (85.6)	28.4	23.9	24.8	27.3	29.0
	Total	3908 (29.1)	2466 (63.1)	19.7	4.4	4.7	3.4	21.7
High Income	Denmark	533 (4.0)	351 (65.9)	2.2	1.1	0.6	0.3	2.9
	Portugal	2514 (18.7)	2066 (82.2)	8.0	0.5	0.9	0.9	8.7
	France	1282 (9.6)	900 (70.2)	7.6	1.8	2.2	1.1	8.9
	Czechia	935 (7.0)	614 (65.7)	10.2	5.1	2.6	2.1	12.5
	Luxembourg	210 (1.6)	121 (57.6)	17.1	1.7	0.8	1.7	16.5
	Panama	521 (3.9)	313 (60.1)	14.2	2.5	2.2	1.7	16.6
	Latvia	139 (1.0)	90 (64.7)	13.8	4.3	1.1	8.3	17.8
	USA	215 (1.6)	167 (77.7)	17.2	1.2	1.8	0.0	18.6
	Sweden	617 (4.6)	420 (68.1)	15.0	8.3	7.1	8.0	19.1
	Uruguay	486 (3.6)	306 (63.0)	16.9	1.9	1.3	1.0	19.6
	Italy	204 (1.5)	127 (62.3)	18.2	1.5	2.1	0.7	19.7
	Australia	379 (2.8)	229 (60.4)	19.2	2.2	3.0	1.7	19.7
	Germany	484 (3.6)	258 (53.3)	18.6	1.6	1.9	1.2	19.8
	Canada	126 (1.0)	91 (72.2)	25.3	2.2	0.0	0.0	25.3
	Singapore	241 (1.8)	95 (39.4)	23.2	20.0	15.8	11.8	27.4
	Total	8886 (66.2)	6148 (69.2)	11.2	2.4	2.1	2.0	12.6
Total		13,416 (100.0)	9004 (67.1)	13.8	3.2	3.0	2.5	15.5

^a^ Those whose partner had insulted them or made them feel bad about themselves

^b^ Those who were made to have unwanted sexual intercourse with their partner by physical force or because they were afraid of what their partner might do

^c^ Those whose partner had slapped, pushed, hit, kicked or choked them or thrown something at them which could cause injury

^d^ Those whose partner restricted their online or telephone contact with family and friends

^e^ Participants who experienced any or multiple of the above forms of violence

^f^ Participants in this country who experienced this form of intimate partner violence, as a proportion of those in the country who responded to the relevant survey item

Table 2 describes the characteristics of the final analysis population, which included 8362 women with complete data on IPV experience and all covariates included in the final model (62.3% of the study population, Appendix G).). Of the 8362 included women, 1.1% were from low-income countries, 30.0% from eight middle-income countries, and 69.0% from 15 high-income countries. The median age was 32 (range 18–83) and the majority were heterosexual (81.4%). Over half had no children (56.3%), and were not married (57.2%), with 61.1% living with a partner during the pandemic, 72.3% had completed some tertiary education, and 68.3% were employed or self-employed prior to the COVID-19 pandemic. Over a third (42.3%) described their household income before COVID-19 as above average in their country.

**Table 2:** Distribution of characteristics of interest among the analysis sample (respondents to the I-SHARE 2020-21 survey with complete data on IPV experience, age, changes to employment status during COVID-19 and their cannabis use, N = 0.8362)

	Characteristic	Frequency (%)
Age (years)		
	18–24	1648 (19.7)
	25–30	2202 (26.3)
	31–40	2459 (29.4)
	> 40	2053 (24.6)
Sexual orientation		
	Heterosexual	6808 (81.4)
	Other sexual orientation ^a, b^	1333 (15.9)
	Missing	221 (2.6)
Ethnicity		
	Majority in country	4852 (58.0)
	Minority in country	603 (7.21)
	Unclear or missing	2907 (34.8)
Educational attainment		
	Primary or less than primary	431 (5.2)
	Secondary (partial or completed)	1512 (18.1)
	University or college (partial or completed)	6044 (72.3)
	Other ^a^	369 (4.4)
	Missing	6 (0.1)
Employment status before the pandemic		
	Employed or self-employed	5709 (68.3)
	Unemployed or informal work	560 (6.7)
	Student	1191 (14.2)
	Other or multiple ^a^	902 (10.8)
Change in employment status during the pandemic		
	Continued in same work and site ^c^	2956 (35.4)
	Working from home (completely or partially)	2888 (34.5)
	Lost employment or working reduced hours	1095 (13.1)
	Other ^a, d^	1423 (17.0)
Transactional sex before the pandemic		
	Never	7880 (94.2)
	Yes	117 (1.4)
	Missing	365 (4.4)
Marital status		
	Not married	4779 (57.2)
	Married	3519 (42.1)
	Missing or unclear	64 (0.8)
Children		
	None	4708 (56.3)
	Any	3653 (43.7)
	Missing	1 (0.0)
Perceived household income before the pandemic		
	Less than average	2326 (27.8)
	Average	1504 (18.0)
	Higher than average	3540 (42.3)
	Missing	992 (11.9)
Living with partner during the pandemic		
	Living with partner	5112 (61.1)
	Not living with partner	2973 (35.6)
	Unclear or missing	277 (3.3)
Household composition during the pandemic		
	Unchanged	6965 (83.3)
	Different to before the pandemic	1278 (15.3)
	Missing	119 (1.4)
Frequency drinking alcohol before the pandemic		
	Never	1785 (21.4)
	Up to 4 times a month	5163(61.7)
	Multiple times a week	1407 (16.8)
	Missing	7 (0.1)
Cannabis use before the pandemic		
	Never	7192 (86.0)
	Up to 4 times a month	897 (10.7)
	Multiple times a week	273 (3.3)
IPV experience in the three months before the pandemic		
	Experienced IPV	1548(18.5)
	Did not experience IPV	6814 (81.5)
Average COVID-19 response stringency experienced prior to completing the survey^e^		
	Low	2593 (31.0)
	Medium	3153 (37.7)
	High	2616 (31.3)
Time spent with social distancing measures in place prior to completing the survey		
	1–6 months	1386 (16.6)
	7–9 months	5518 (66.0)
	10–12 months	1458 (17.4)
Total		8362 (100.0)

^a^ Participants selecting “other” for any item were unable to specify their meaning

^b^ Includes participants identifying as lesbian, gay, bisexual, questioning or unsure, asexual, pansexual or other

^c^ Including those who were retired before the COVID-19 pandemic

^d^ Includes participants who have changed job, are employed but unable to work, or who selected other

^e^ Calculated using a summary of the number and intensity of pandemic containment policies experienced by the participant over the period of COVID-19 social distancing

Most had experienced some employment disruption during the pandemic, with 13.1% becoming unemployed or working on reduced hours, and 34.5% working from home. Participants who were unemployed or in informal employment prior to the COVID-19 pandemic were more likely to be unemployed or work in reduced hours during the pandemic (43.1%) than participants who were employed or self-employed before the pandemic (11.7%). In contrast, informally employed and unemployed participants were substantially less likely to be working from home during the pandemic (6.2% compared to 39.9%) (Appendix H). Working from home was more prevalent among upper-middle-income and high-income countries (37.0% and 33.2% respectively) than low and lower-middle-income countries (16.8% and 20.0% respectively) (Appendix I).

Of those working in-person, 12.0% experienced some form of IPV during COVID-19 (Table 3), compared to 15.8% of those working from home, 20.8% of those who lost work, and 17.5% of those who experienced other work disruptions. After adjustment for confounders, there was strong evidence that women working from home experienced greater odds of IPV than those working on-site (aOR 1.40, 95% CI 1.12–1.74, p = 0.003). In contrast, there was poor evidence that losing employment was associated with higher odds of IPV in this period (aOR 1.15, 95% CI 0.87–1.53, p = 0.313). Employment changes in the “other” category were also associated with 46% higher odds of IPV (aOR 1.46, 95% CI 1.11–1.94, p = 0.007). Potential confounders tested included socio-demographic variables, variables related to IPV risk factors prior to COVID-19, and variables related to the stringency and experience of lockdown. After using a forwards modelling approach to identify which adjustment variables did not change the overall effect estimate, the final adjustment set included age, country and sex as a priori confounders, and cannabis use and IPV experience prior to COVID-19.

**Table 3:** Association between changes in employment status and experiencing intimate partner violence outcomes during COVID-19 social distancing measures among cis-gendered women who participated in the I-SHARE survey 2020-21

	Change in employment status	Individuals experiencing outcome (%)	Model 1: OR adjusted for country (95% CI)^a^	Model 2: OR adjusted for confounders (95% CI)^b^	Model 2 Wald test P-value
Experience of physical,sexual or psychological IPV	Continued in same work and site/ retired^c^	376 (12.0)	1	1	
	Working (completely or partially) from home^d^	482 (15.8)	1.24 (1.06–1.44)^h^	1.40 (1.12–1.74)^h^	0.003
	Unemployed or reduced working hours^e^	250 (20.8)	1.66 (1.37-2.00)^h^	1.15 (0.87–1.53)^h^	0.313
	Other^f,g^	274 (17.5)	1.47 (1.22–1.76)^h^	1.46 (1.11–1.94)^h^	0.007
Experience of physical or sexual IPV	Continued in same work and site/ retired^c^	118 (3.7)	1	1	
	Working (completely or partially) from home^d^	130 (4.2)	1.08 (0.82–1.42)^i^	1.20 (0.84–1.72)^i^	0.322
	Unemployed or reduced working hours^e^	85 (7.0)	1.74 (1.27–2.39)^i^	1.32 (0.86–2.04)^i^	0.209
	Other^f,g^	83 (5.3)	1.28 (0.93–1.75)^i^	1.09 (0.70–1.70)^i^	0.690
Experience of psychological violence	Continued in same work and site/ retired^c^	357 (11.3)	1	1	
	Working (completely or partially) from home^d^	458 (14.8)	1.21 (1.03–1.41)^j^	1.35 (1.07–1.69)^j^	0.003
	Unemployed or reduced working hours^e^	235 (19.2)^f^	1.61 (1.33–1.95)^j^	1.16 (0.87–1.54)^j^	0.299
	Other^f,g^	251 (15.9)^g^	1.38 (1.14–1.66)^j^	1.37 (1.02–1.83)^j^	0.025

^a^ Not including individuals with missing data on any variables in model 2 (the fully adjusted model), to allow for direct comparison

^b^ Adjusted for country, age in 4 levels, whether the participant experienced the relevant IPV outcome in the three months before COVID-19, employment status before COVID-19 and cannabis use frequency before COVID-19 measures

^d c^ N = 2956

^d^ N = 2888

^e^ N = 1095

^f^ N = 1423

^g^ Includes those who changed job, were paid but unable to work, or answered “other”. Those selecting “other” were unable to specify further

^h^ N = 8362

^i^ N = 8467

^j^ N = 8513

The results were similar for women in HICs, and LMICs (Appendix J). The association between working from home and IPV was stronger in countries with higher gender inequality (aOR 1.51, 95% CI 1.11–2.06) compared to more equal societies (aOR 1.26, 95% CI 0.91–1.76), measured using the 2019 United Nations Development Programme Gender Inequality Index (Appendix K) [26]. There remained no evidence of an association between losing work and IPV after stratifying by country-level gender inequality. The estimated effect of working from home on IPV was greater among women living with a partner than not living with a partner (Appendix L), although there was not strong statistical support for an interaction (estimated interaction term 1.31 (0.95–1.82, p = 0.100).

**Fig. 2:**
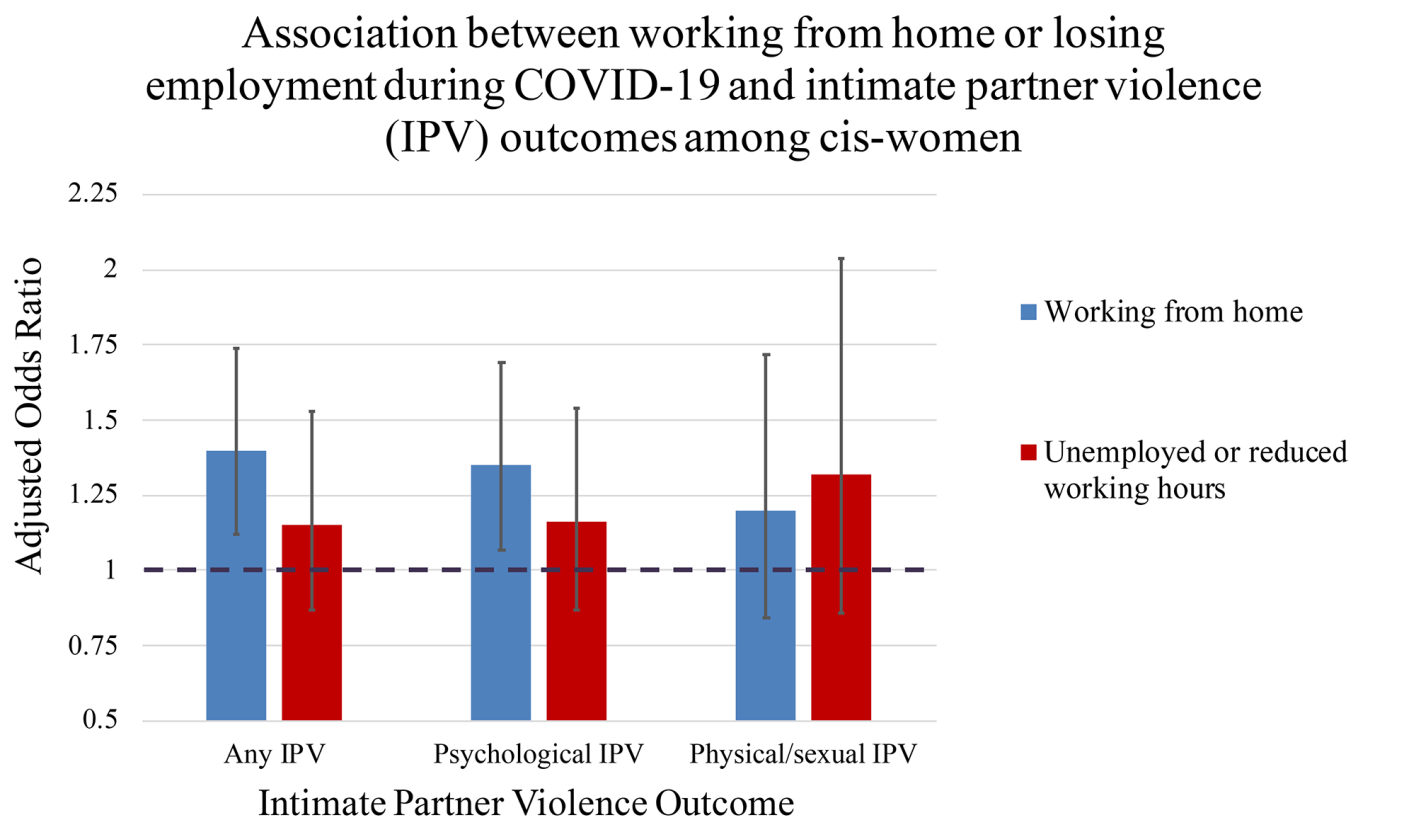
Association between employment changes during COVID-19 and intimate partner violence among cis-women Legend: Association between different forms of intimate partner violence experienced during COVID-19 social distancing measures, and working from home or losing employment during this period, amongst cis-gendered women who participated in the I-SHARE 2020-21 survey and responded to variables included in the model (N = 8362). Those who continued in the same work and site or were retired during the pandemic are the reference group. The estimates are adjusted for country, age group, whether the participant experienced IPV in the three months before COVID-19, employment status before COVID-19 and cannabis use frequency before COVID-19 measures. The black bars show the 95% confidence intervals

When the outcome is disaggregated into physical/sexual IPV and psychological IPV (Fig. 2), the associations with psychological IPV were similar to the estimated associations with any IPV (Table 3). There was weak statistical support for an association between physical/sexual IPV and home working (aOR 1.20, 95% CI 0.84–1.72, p = 0.322); or physical/sexual IPV and loss of work (aOR 1.32, 95% CI 0.86–2.04, p = 0.209).

32.9% of participants had missing data on at least one IPV item during COVID-19. 67.3% of these participants identified as single and 76.3% identified as single, widowed or divorced, and so may not have perceived the questions as relevant. Participants were more likely to have responded to all IPV items if they reported being heterosexual, being in a relationship, being in the majority ethnicity in their country, being in a higher income group, and not experiencing IPV before COVID-19 (Appendix M). The results were robust after multiple imputation for the outcome, imputed using country and relationship status (Appendix E). The results were also robust after restriction to the probability-based samples in Kenya, Botswana, Sweden, Argentina, Denmark and Czechia (Appendix N), and after including those with partial responses to the outcome (Appendix O).

## Discussion

This analysis presents evidence that working from home during COVID-19 measures was associated with a 40% increase in odds of IPV compared to working in-person. We found no relationship between losing employment and IPV risk, but strong evidence that those who experienced other disruptions to work, including changing job, or being paid but unable to work, experienced higher IPV. This extends the literature on IPV during COVID-19 by leveraging a large, multi-country study. These findings have important implications for how an employer’s duty of care extends to the domestic environment for staff who are working from home.

Amongst this sample of cis-gender women, 15.5% experienced IPV during the initial COVID-19 wave. This is consistent with single-country analyses of IPV during COVID-19 [27, 28]. The majority of these women had also experienced IPV in the three months prior to the pandemic, indicating a persistent exposure to violence. This substantial burden of IPV has implications for women, families, and health systems around the world. This underscores the need for more resources focused on groups of women who may have substantial unmet IPV needs.

Our data suggest that women who worked from home during COVID-19 were more likely to experience IPV. Odds of experiencing physical, sexual, or psychological IPV were all elevated compared to those working in-person, although statistical evidence for an association between working from home and physical/sexual violence was weaker. Possible explanations for the elevated risk of IPV amongst women working from home include increased time exposed to a partner at home and the removal of workplace support structures, including social contacts and a place from which to access support [16, 17]. Furthermore adapting to working from home may have heightened relational tension, particularly among couples attempting to additionally manage childcare and home-schooling [13]. The association was stronger in countries with higher gender inequality, where violence is more often perceived as an acceptable response to relational pressures [3].

The association is consistent with a smaller study in the United States which estimated that IPV increased as a result of people staying-at home during COVID-19 [29]. Several pre-COVID-19 studies suggest working outside the home was associated with increased physical and emotional IPV amongst women in India [30], Mexico [31], and South America [32, 33]. However, the studies found conflicting impacts on sexual IPV, and it was unclear whether the comparator “working from home” group in these studies was performing paid work or not.

We found that there was no relationship between losing employment and experiencing IPV during COVID-19. This finding was unexpected because unemployment has been found to be associated with IPV in other settings [13, 28]. It is possible that when a participant loses work and becomes more economically dependent on a partner, the partner feels they have gained control and may perceive less need to use violence [13]. For couples with children, loss of work for one person might reduce the stress of balancing work and childcare while schools were closed. Emergency COVID support measures may also have buffered the economic shock of losing employment. Furthermore, women who earn more than a male partner are at greater IPV risk in cultures where men are expected to provide for the family [4, 34]. During the pandemic this could place women in heterosexual relationships who continue working at greater risk of IPV than those who lose employment, particularly if their partner’s work is disrupted.

A strength of the study was that using an online survey allowed a large international sample to be reached rapidly, despite social contact and travel restrictions. The study benefited from using locally driven surveys and having a pre-specified focus on IPV as a primary outcome. IPV was measured using a validated format which, alongside the anonymity of an online survey, promotes IPV disclosure and facilitates comparison with other IPV surveys [24].

The following limitations must be considered when interpreting these results. First, the cross-sectional design means some IPV experiences could have preceded working from home. We speculate that this alternative explanation is less likely because many women started to work from home early in the first wave. A second key limitation is that most countries relied on convenience sampling. This meant the proportion of childless and university educated women was high, which may limit generalisability to countries with lower education rates, and introduce collider bias effects. However, the results were robust to restricting the analysis to the population-representative or quota-based samples, which are less susceptible to selection bias. Thirdly, in all countries only internet users, who are more likely to be young and highly educated than non-users were able to participate, and women whose internet access was monitored by a controlling partner may have been unable or unsafe to participate. We therefore expect IPV prevalence to be underestimated in this study. Fourth, one third of participants had missing data on at least one IPV item during COVID-19. Of these non-responders, 76.3% identified as single, widowed or divorced so may not have perceived the questions as relevant. Fifth, partner characteristics were not measured, but are important for understanding the context of IPV. For example, relational tension and exposure to an abusive partner could be exacerbated if the partner’s work is also disrupted. Finally, although the overall number of participants is large, few were from low-income countries. Since resilience to employment shocks is lower in developing countries, and working from home is less common in these locations (Appendix I), the association with IPV may differ in these settings [35].

Understanding risk factors for IPV is an important step in developing interventions to prevent violence. A review of reviews revealed that successful interventions aim to address IPV risk factors, including societal expectations around violence and gender relations, as well as providing support to survivors [36]. Interventions are most promising when they adopt a multisectoral and community-based approach, incorporating elements of group training, public events, digital campaigns, and economic transfers [36, 37]. WHO recommendations further emphasise the importance of cross-sectoral responses to IPV during the COVID-19 pandemic [38]. Our findings indicate that employers can be an additional important stakeholder in this field, and that programmes to address violence should consider including interventions related to employment alongside other IPV risk factors. Furthermore, it is critical to ensure that programmes to address violence, whether at a community or individual level, do not neglect women who may be isolated at home, both during and beyond pandemic contexts.

## Conclusions

In conclusion this analysis provides evidence that working from home during COVID-19 was associated with higher IPV during the pandemic among cis-gender women. During COVID-19 restrictions or other pandemics, workplaces should work with legal, health and social services to strengthen support offered to staff at risk of IPV [39]. Yet, offering the choice to attend a workplace in person can be a source of resiliency [16, 17]. Today businesses, governments, and other organizations are considering how to make working from home a more permanent feature of work. Our findings have important implications for these decisions. Future research could explore whether this resiliency can be transferred to those working at home through employer interventions, during and outside of pandemic circumstances.

## Electronic supplementary material

Below is the link to the electronic supplementary material.

Supplementary Material 1

## Data Availability

Data sharing agreements govern access to the data necessary for multi-country comparisons within the I-SHARE research consortium. In-country leads have oversight for country-level data, and regulate access to this information. More information, including the protocol and data management plan are available on the I-SHARE website (https://ishare.web.unc.edu). Information on restriction stringency from the Oxford COVID-19 Government Response Tracker is publicly available at the following link: https://github.com/OxCGRT/covid-policy-tracker. Information on gender equality at a country level (2019) is available in the UNDP Human Development Reports data repository, in the table “All composite indices and components time series (1990–2021)”, available at: https://hdr.undp.org/data-center/documentation-and-downloads. The STATA do-file used for this analysis are available on request to the corresponding author (Naomi Miall).

## List of Abbreviations

aOR: Adjusted Odds Ratio
95% CI: Confidence Interval
HIC: High Income Country
IPV: Intimate partner violence
I-SHARE: International Sexual Health and Reproductive Health Survey
LMIC: Low or Middle Income Country
OX-CGRT: Oxford COVID-19 Government Response Tracker
WHO: World Health Organisation
