# Supplementary material for: Working from home and intimate partner violence among cis-women during the COVID-19 pandemic: evidence from a global, cross-sectional study
Source: BMC Public Health. 2023 May 26;23:965. doi: 10.1186/s12889-023-15785-7 (PMC10214313; doi:10.1186/s12889-023-15785-7)
Supplement: Supplementary file 1 — Supplementary Material 1 [file 12889_2023_15785_MOESM1_ESM.docx]

**Appendices**

**A Sampling method used and number of participants in countries involved in the I-SHARE survey**

**Table A:** Countries using convenience sampling, population-representative sampling, or online panels in the I-SHARE 2020-21 survey, and the number of participants included in the study population for this study.

| **Sampling method** | **Countries in the I-SHARE study using this sampling method** | **Sample size (%)** |
| --- | --- | --- |
| **Convenience** | Egypt^1^ | 0 (0.0) |
|  | South Africa^1^ | 0 (0.0) |
|  | Spain^1^ | 0 (0.0) |
|  | Mozambique | 35 (0.3) |
|  | Malaysia | 112 (0.8) |
|  | Canada | 126 (1.0) |
|  | Nigeria | 129 (1.0) |
|  | Latvia | 139 (1.0) |
|  | China | 153 (1.1) |
|  | Moldova | 189 (1.4) |
|  | Italy | 204 (1.5) |
|  | Luxembourg | 210 (1.6) |
|  | USA | 215 (1.6) |
|  | Singapore | 241 (1.8) |
|  | Czechia | 378 (2.8) |
|  | Australia | 379 (2.8) |
|  | Germany | 484 (3.6) |
|  | Uruguay | 486 (3.6) |
|  | Panama | 521 (3.9) |
|  | Mexico | 1206 (9.0) |
|  | France | 1282 (9.6) |
|  | Colombia | 1482 (11.1) |
|  | Portugal | 2514 (18.7) |
|  | **Total** | **10485 (78.2)** |
| **Online panels** | Lebanon^1^ | 0 (0.0) |
|  | Uganda | 108 (0.8) |
|  | Kenya | 161 (1.2) |
|  | Botswana | 278 (2.1) |
|  | Sweden | 617 (4.6) |
|  | Argentina | 677 (5.1) |
|  | **Total** | **1841 (13.7)** |
| **Population representative** | Denmark | 533 (4.0) |
|  | Czechia | 557 (4.2) |
|  | **Total** | **1090 (8.1)** |
| Total | | 13416 (100.0) |

^1^Countries excluded from analysis due to participant numbers <20: Spain [2]; Lebanon [10]; South Africa [12]; Egypt [17].

**B Exploring sensitivity of results to including women in countries with less than 200 participants.**

**Table B:** Association between changes in employment status and experiencing intimate partner violence (IPV) during COVID-19 social distancing measures among cis-gender women who participated in the I-SHARE survey 2020-21, including 41 women from countries with less than 20 participants.

^a^ Not including individuals with missing data on any variables in model 2 (the fully adjusted model), to allow for direct comparison.

^b^ Adjusted for country, age in 4 levels, whether the participant experienced the relevant IPV outcome in the three months before COVID-19, employment status before COVID-19 and cannabis use frequency before COVID-19 measures.

^C^ Includes those who changed job, were paid but unable to work, or answered “other”. Those selecting “other” were unable to specify further.

^e^ N=8377

^f^ N=8477

^g^N=8528

|  | **Change in employment status** | **Individuals experiencing outcome (%)** | **Model 1: OR adjusted for country**  **(95% CI)^a^** | **Model 2: OR adjusted for confounders (95% CI)^b^** | **Model 2 Wald test P-value** |
| --- | --- | --- | --- | --- | --- |
| Experience of physical,sexual or psychological IPV | Continued in same work and site/ retired | 377 (12.1) | 1 | 1 |  |
|  | Working (completely or partially) from home | 483 (15.8) | 1.23 (1.06-1.44)^e^ | 1.40 (1.12-1.74)^e^ | 0.003 |
|  | Unemployed or reduced working hours | 253 (20.9) | 1.66 (1.37-2.00)^e^ | 1.16 (0.88-1.53)^e^ | 0.306 |
|  | Other^c^ | 274 (17.5) | 1.46 (1.22-1.76)^e^ | 1.46 (1.11-1.93)^e^ | 0.007 |
| Experience of physical or sexual IPV | Continued in same work and site/ retired | 118 (3.7) | 1 | 1 |  |
|  | Working (completely or partially) from home | 131 (4.2) | 1.09 (0.83-1.43)^f^ | 1.21 (0.85-1.74)^f^ | 0.293 |
|  | Unemployed or reduced working hours | 86 (7.1) | 1.75 (1.28-2.39)^f^ | 1.29 (0.83-1.99)^f^ | 0.254 |
|  | Other^c^ | 83 (5.3) | 1.28 (0.93-1.75)^f^ | 1.08 (0.69-1.67)^f^ | 0.739 |
| Experience of psychological violence | Continued in same work and site/ retired | 358 (11.3) | 1 | 1 |  |
|  | Working (completely or partially) from home | 459 (14.8) | 1.20 (1.03-1.41)^g^ | 1.35 (1.07-1.69)^g^ | 0.012 |
|  | Unemployed or reduced working hours | 238 (19.3) | 1.61 (1.33-1.95)^g^ | 1.16 (0.87-1.55)^g^ | 0.315 |
|  | Other^c^ | 251 (15.8) | 1.37 (1.14-1.65)^g^ | 1.37 (1.02-1.83)^g^ | 0.035 |

**C I-SHARE survey items measuring IPV during COVID-19**

**Table C:** Survey items used in the I-SHARE 2020-21 survey to measure intimate partner violence, and the type of violence they relate to.

| **Type of intimate partner violence** | **Survey question**  “During COVID-19 social distancing measures, has a partner… |
| --- | --- |
| Psychological (controlling behaviour) | Tried to restrict (online or phone) contact with your family?”^a^ |
| Psychological (emotional) | Insulted you or made you feel bad about yourself?”^a^ |
| Physical | Slapped, pushed, hit, kicked or choked you or thrown something at you that could hurt you?”^b^ |
| Sexual | Physically forced you to have sexual intercourse when you did not want to?”^a^ |
| Sexual | Made you have sexual intercourse when you did not want to because you were afraid of what your partner might do?”^a^ |

^a^ Questions are part of the validated WHO instrument.

^b^ Question combines several items in the validated WHO instrument, which asks about these behaviours individually.

**D Data processing**

**Table D:** Survey items used in the I-SHARE 2020-21 survey to measure variables used in this analysis.

| **Variable** | **Survey item** | **Response options** |
| --- | --- | --- |
| Gender | What sex were you assigned at birth, on your original birth certificate? | Male |
|  |  | Female |
|  |  | Other |
|  | Which of the following do you currently identify as? | Cisgender |
|  |  | Non-cisgender |
|  |  | Other |
| Relationship status | Which best describes your relationship status? | Single and never had a partner |
|  |  | Single but had a partner previously |
|  |  | In a relationship but not living together |
|  |  | Not legally married but living with a partner |
|  |  | Legally married and living together |
|  |  | Legally married and not living together |
|  |  | Legally married but separated |
|  |  | Widowed |
|  |  | Divorced |
|  |  | Other |
| IPV experience during the COVID-19 lockdown | During the COVID-19 social distancing measures ….   1. Has a partner tried to restrict (online or phone) contact with your family? 2. Has a partner insulted you or made you feel bad about yourself? 3. Has a partner slapped, pushed, hit, kicked or choked you or thrown something at you that could hurt you? 4. Has a partner physically forced you to have sexual intercourse when you did not want to? 5. Has a partner made you have sexual intercourse when you did not want to because you were afraid of what your partner might do? | No  Yes, once^a^  Yes, multiple times^a^ |
| Employment disruption during COVID-19 | Since the COVID-19 social distancing measures, has your employment status changed? | No change: I continue doing the same work and going to the usual job site |
|  |  | I keep doing the same work, but (partially) from home |
|  |  | I work on reduced time^a^ |
|  |  | I lost my job/work/business^a^ |
|  |  | I am temporarily unemployed^a^ |
|  |  | I am employed and paid but unable to attend or do work^b^ |
|  |  | I changed work/jobs^b^ |
|  |  | Other^b^ |

**Continued…**

**Table D continued:**

| **Variable** | **Survey item** | **Response options** |
| --- | --- | --- |
| Age | How old are you? | Free text |
| Sexual orientation | What is your sexual orientation? | Heterosexual |
|  |  | Bisexual^a^ |
|  |  | Gay^a^ |
|  |  | Lesbian^a^ |
|  |  | Pansexual^a^ |
|  |  | Asexual^a^ |
|  |  | Questioning or unsure^a^ |
|  |  | Other^a^ |
| Ethnicity | What is your ethnicity, origin group or caste? | Free text |
| Educational attainment | What is your highest degree of schooling? | No formal education^a^ |
|  |  | Some primary school^a^ |
|  |  | Completed primary school^a^ |
|  |  | Some secondary school^b^ |
|  |  | Completed secondary school^b^ |
|  |  | Some college or university^c^ |
|  |  | Completed college or university^c^ |
|  |  | Other |
| Employment status before COVID-19 | What was your employment status the month before the COVID-19 social distancing measures? | Employed and received a salary^a^ |
|  |  | Self-employed / Business owner^a^ |
|  |  | Unemployed^b^ |
|  |  | Informal / Piecemeal work^b^ |
|  |  | Student |
|  |  | Retired / Pensioned^c^ |
|  |  | Other^c^ |
| Transactional sex | In the three months before the COVID-19 social distancing measures, how many times have you had sex in exchange for money, material goods, favors, drugs, or shelter? | Never |
|  |  | Monthly or less^a^ |
|  |  | 2-4 times a month^a^ |
|  |  | 2-3 times a week^a^ |
|  |  | 4 or more times a week^a^ |
| Marital status | Which best describes your relationship status? | Legally married and living together^a^ |
|  |  | Legally married and not living together^a^ |
|  |  | Legally married but separated^a^ |
|  |  | Single and never had a partner^b^ |
|  |  | Single but had a partner previously^b^ |
|  |  | In a relationship but not living together^b^ |
|  |  | Not legally married but living with a partner^b^ |
|  |  | Widowed^b^ |
|  |  | Divorced^b^ |
|  |  | Other |
| Children | How many children do you have, if any? | Free text |

**Continued…**

**Table D continued:**

| **Variable** | **Survey item** | **Response options** |
| --- | --- | --- |
| Living with a partner during COVID-19 | Which best describes your relationship status? | Not legally married but living with a partner^a^ |
|  |  | Legally married and living together^a^ |
|  |  | Single and never had a partner^b^ |
|  |  | Single but had a partner previously^b^ |
|  |  | In a relationship but not living together^b^ |
|  |  | Legally married and not living together^b^ |
|  |  | Legally married but separated^c^ |
|  |  | Widowed^c^ |
|  |  | Divorced^c^ |
|  |  | Other^c^ |
| Household income before COVID-19 | Below is an income scale on which 1 indicates the lowest income group and 10 the highest income group in your country. We would like to know in what group your household was in the year before the COVID-19 crisis? | 0-10 |
| Alcohol use before COVID-19 | How often did you have a drink containing alcohol before the COVID-19 social distancing measures? | Never |
|  |  | Monthly or less^a^ |
|  |  | 2-4 times a month^a^ |
|  |  | 2-3 times a week^b^ |
|  |  | 4 or more times a week^b^ |
| Cannabis use frequency before COVID-19 | How often did you use cannabis (marijuana, hash, grass) before the COVID-19 social distancing measures? | Never |
|  |  | Monthly or less^a^ |
|  |  | 2-4 times a month^a^ |
|  |  | 2-3 times a week^b^ |
|  |  | 4 or more times a week^b^ |
| IPV experience in the three months before COVID-19 | In the three months before the COVID-19 social distancing measures…   1. Has a partner tried to restrict (online or phone) contact with your family? 2. Has a partner insulted you or made you feel bad about yourself? 3. Has a partner slapped, pushed, hit, kicked or choked you or thrown something at you that could hurt you? 4. Has a partner physically forced you to have sexual intercourse when you did not want to? 5. Has a partner made you have sexual intercourse when you did not want to because you were afraid of what your partner might do? | No |
|  |  | Yes, once^a^ |
|  |  | Yes, multiple times^a^ |

^a,b,c^ Within each variable, responses that were grouped together are marked with the same superscript.

**E Exploring sensitivity of results to missing outcome data**

Multiple imputation was used to explore the sensitivity of the results to missing data about IPV during COVID-19 social distancing. Since data was available on the pre-COVID employment status, country, and date of completion for all participants, and these variables were associated with IPV, a set of 10 datasets were generated, each with a value filling in the missing data points drawn from a logistic probability distribution based on the known values. The estimates from each dataset are averaged, taking account of the extra variability of the imputation process, to generate an overall effect size. This process makes the assumption that the data are missing at random, meaning that after accounting for country and pre-COVID employment status and date, the chance of the data on IPV being missing is independent of its value. This is a strong assumption since country, date and prior employment status are unlikely to perfectly predict IPV, and it is likely that whether the data is missing does depend on its value.

**Table E:** Association between changes in employment status and experiencing intimate partner violence (IPV) during COVID-19 social distancing measures among cis-gender women who participated in the I-SHARE survey 2020-21, using complete records analysis or using multiple imputation to handle missing data on IPV experience during COVID-19.

| **Change in employment status** | **Fully adjusted OR estimates in main analysis (excluding those with missing data) (95% CI)^a^** | **Fully adjusted OR estimates after multiple imputation for missing data on IPV during COVID (95% CI) ^a, b^** | **Wald test p-value** |
| --- | --- | --- | --- |
| Continued in same work and site/ retired | 1 | 1 |  |
| Working (completely or partially) from home | 1.40 (1.12-1.74) | 1.30 (1.07-1.57) | 0.008 |
| Unemployed or reduced working hours | 1.15 (0.87-1.53) | 1.08 (0.84-1.40) | 0.531 |
| Other ^c^ | 1.46 (1.11-1.94) | 1.31 (1.03-1.67) | 0.030 |

^a^ Adjusted for country, age in 4 levels, whether the participant experienced IPV in the three months before COVID, employment status before COVID-19 measures, and cannabis use frequency before COVID-19 measures.

^b^ Imputed values for IPV experience for those who did not respond to this question drawn from a logistic probability model based on the participant’s country, date of participation and prior employment status.

^c^ Includes those who those who changed job, were paid but unable to work or answered “other”. Those who selected “other” were unable to specify further.

**F Association between IPV experience prior to and during the COVID-19 pandemic**

**Table F**: Proportion of those who experienced IPV during the COVID-19 lockdown in their country, that also experienced IPV in the three months prior to the lockdown.

|  | **Did not experience IPV in the 3 months prior to pandemic restrictions (%)** | **Experienced IPV in the 3 months prior to pandemic restrictions (%)** | **Did not respond to questions about IPV experience prior to pandemic restrictions (%)** | **Total (%)** |
| --- | --- | --- | --- | --- |
| **Experienced IPV during pandemic restrictions** | 212  (15.2) | 1114  (80.0) | 66  (4.7) | 1392  (100) |
| **Did not experience IPV during pandemic restrictions** | 6713  (88.2) | 486  (6.4) | 413  (5.4) | 7612  (100) |

**G Flow diagram indicating the composition of the study population and analysis population**

**H** **Distribution of changes to employment experienced during the COVID-19 pandemic by employment status prior to the pandemic.**

**Table H**: Comparison of the prevalence of employment pattern changes during COVID-19, between study participants with different employment types before the COVID-19 lockdown.

| **Employment status before COVID-19** | **Continued in same work and site/ retired (%)** | **Working (completely or partially) from home (%)** | **Unemployed or reduced working hours**  **(%)** | **Other^a,b^**  **(%)** | **Missing** | **Total**  **(%)** |
| --- | --- | --- | --- | --- | --- | --- |
| Employed or self-employed | 3352  (39.4) | 3392  (39.9) | 996  (11.7) | 749  (8.8) | 9  (0.1) | 8498  (100) |
| Student | 326  (13.6) | 830  (34.6) | 295  (12.3) | 889  (37.1) | 59  (2.5) | 2399  (100) |
| Informal work or unemployed | 115  (11.5) | 62  (6.2) | 432  (43.1) | 374  (37.3) | 19  (1.9) | 1002  (100) |
| Other^b^ or multiple employment types | 614  (40.5) | 230  (15.2) | 216  (14.2) | 449  (29.6) | 8  (0.5) | 1517  (100) |

^a^ Includes those who those who changed job, were paid but unable to work or answered “other”.

^b^ Those who selected “other” on either question were unable to specify further.

**I Distribution of the exposure changes in employment status by country income**

**Table I**: The proportion of participants from low income, lower middle income, upper middle income, and high-income countries who experienced each change in employment category during the COVID-19 pandemic.

| **World Bank Economy Classification** | **Continued in same work and site/ retired (%)** | **Working (completely or partially) from home (%)** | **Unemployed or reduced working hours**  **(%)** | **Other^a^**  **(%)** | **Missing** | **Total**  **(%)** |
| --- | --- | --- | --- | --- | --- | --- |
| Low Income Country | 55  (38.5) | 24  (16.8) | 42  (29.4) | 17  (11.9) | 5  (3.5) | 143  (100) |
| Lower Middle Income Country | 162  (33.8) | 96  (20.0) | 102  (21.3) | 115  (24.0) | 4  (0.8) | 479  (100) |
| Upper Middle Income Country | 967  (24.7) | 1447  (37.0) | 658  (16.8) | 813  (20.8) | 23  (0.6) | 3908  (100) |
| High Income Country | 3223  (36.3) | 2947  (33.2) | 1137  (12.8) | 1516  (17.1) | 63  (0.7) | 8886  (100) |

^a^ Includes those who those who changed job, were paid but unable to work or answered “other”. Those who selected “other” were unable to specify further.

**J Results stratified into low and middle income country or high income country**

**Table J:** Association between changes in employment status and experiencing intimate partner violence during COVID-19 social distancing measures among cis-gender women who participated in the I-SHARE survey 2020-21, organised according to the World Bank economy classification.

| **World Bank Economy Classification** | **Change in employment status** | **Individuals experiencing IPV (%)** | **OR adjusted for country**  **(95% CI)^a^** | **Fully adjusted OR**  **(95% CI)^b^** | **Wald test p-value** |
| --- | --- | --- | --- | --- | --- |
| Low or Middle Income Country | Continued in same work and site/ retired | 145 (18.6) | 1 | 1 |  |
|  | Working (completely or partially) from home | 228 (22.1) | 1.26 (0.99-1.61)^c^ | 1.62 (1.13-2.32)^c^ | 0.008 |
|  | Unemployed or reduced working hours | 119 (25.0) | 1.50 (1.12-2.01)^c^ | 1.06 (0.68-1.63)^c^ | 0.799 |
|  | Other ^e^ | 120 (21.6) | 1.30 (0.97-1.73)^c^ | 1.64 (1.03-2.62)^c^ | 0.038 |
| High Income Country | Continued in same work and site/ retired | 231 (9.9) | 1 | 1 |  |
|  | Working (completely or partially) from home | 254 (12.5) | 1.16 (0.95-1.41)^d^ | 1.25 (0.94-1.66)^d^ | 0.126 |
|  | Unemployed or reduced working hours | 131 (18.0) | 1.75 (1.37-2.24)^d^ | 1.28 (0.89-1.85)^d^ | 0.186 |
|  | Other ^e^ | 154 (15.2) | 1.58 (1.26-1.99)^d^ | 1.40 (0.98-1.99)^d^ | 0.061 |

^a^ Not including individuals with missing data on any of the variables in the fully adjusted model for this variable, to allow for direct comparison.

^b^ Adjusted for country, age in 4 levels, whether the participant experienced IPV in the three months before COVID, employment status before COVID-19 measures, and cannabis use frequency before COVID-19 measures.

^c^ N= 2745 from 10 countries. Note Kenya excluded due to missing data on age and cannabis use.

^d^ N=5766 from 15 countries.

^e^ Includes those who those who changed job, were paid but unable to work or answered “other”. Those who selected “other” were unable to specify further.

**K Results stratified into countries with high and low gender inequality**

**Table K:** Association between changes in employment status and experiencing intimate partner violence during COVID-19 social distancing measures among cis-gender women who participated in the I-SHARE survey 2020-21, organised according to the United Nations Development Programme (UNDP) 2019 Gender Inequality Index (GII) score of their country of residence.

| **UNDP Gender Inequality Index score^a^** | **Change in employment status** | **Individuals experiencing outcome (%)** | **Model 1: OR adjusted for country**  **(95% CI)^d^** | **Model 2: OR adjusted for confounders (95% CI)^e^** | **Model 2 Wald test P-value** |
| --- | --- | --- | --- | --- | --- |
| Low gender inequality^b^ | Continued in same work and site/ retired | 185 (8.9) | 1 | 1 |  |
|  | Working (completely or partially) from home | 200 (11.7) | 1.19 (0.95-1.48)^f^ | 1.26 (0.91-1.76)^f^ | 0.163 |
|  | Unemployed or reduced working hours | 99 (17.3) | 1.85 (1.40-2.44)^f^ | 1.20 (0.78-1.83)^f^ | 0.410 |
|  | Other^d^ | 130 (14.9) | 1.73 (1.35-2.23)^f^ | 1.60 (1.07-2.39)^f^ | 0.023 |
| High gender inequality^c^ | Continued in same work and site/ retired | 188 (18.2) | 1 | 1 |  |
|  | Working (completely or partially) from home | 280 (21.2) | 1.28 (1.03-1.60)^g^ | 1.51 (1.11-2.06)^g^ | 0.009 |
|  | Unemployed or reduced working hours | 150 (24.0) | 1.52 (1.17-1.98)^g^ | 1.16 (0.80-1.69)^g^ | 0.434 |
|  | Other^d^ | 144 (21.0) | 1.28 (0.98-1.66)^g^ | 1.44 (0.96-2.16)^g^ | 0.075 |

^a^ Gender inequality measures gender inequality in reproductive health, empowerment and the labour market. Available at <https://hdr.undp.org/data-center/documentation-and-downloads>

^b^ GII score less than 0.136 (the median): Australia; Canada; Czechia; Denmark; France; Germany, Italy; Luxembourg; Portugal; Singapore; Sweden.

^c^ GII score more than 0.136: Argentina; Botswana; China; Colombia; Kenya; Latvia; Malaysia; Mexico; Moldova; Mozambique; Panama: Uganda: Uruguay: USA.

^d^ Not including individuals with missing data on any variables in model 2 (the fully adjusted model), to allow for direct comparison.

^e^ Adjusted for country, age in 4 levels, whether the participant experienced the relevant IPV outcome in the three months before COVID-19, employment status before COVID-19 and cannabis use frequency before COVID-19 measures.

^f^ Includes those who changed job, were paid but unable to work, or answered “other”. Those selecting “other” were unable to specify further.

^g^ N=4907

^h^ N=3412

**L Results stratified by living situation**

**Table L:** Association between changes in employment status and experiencing intimate partner violence during COVID-19 social distancing measures among cis-gender women who participated in the I-SHARE survey 2020-21, stratified according to whether the women are living with a partner or not.

| **Living situation** | **Change in employment status** | **Individuals experiencing IPV (%)** | **OR adjusted for country**  **(95% CI)^a^** | **Fully adjusted OR**  **(95% CI)^b^** | **Wald test p-value** |
| --- | --- | --- | --- | --- | --- |
| Not living with a partner | Continued in same work and site/ retired | 130 (12.1) | 1 | 1 |  |
|  | Working (completely or partially) from home | 147 (13.9) | 1.12 (0.85-1.47)^d^ | 1.22 (0.85-1.76)^d^ | 0.283 |
|  | Unemployed or reduced working hours | 88 (18.2) | 1.35 (0.98-1.87)^d^ | 1.23 (0.79-1.91)^d^ | 0.371 |
|  | Other ^c^ | 116 (16.1) | 1.16 (0.85-1.57)^d^ | 1.32 (0.85-2.06)^d^ | 0.213 |
| Living with a partner | Continued in same work and site/ retired | 231 (11.9) | 1 | 1 |  |
|  | Working (completely or partially) from home | 311 (16.4) | 1.25 (1.03-1.52)^e^ | 1.42 (1.06-1.91)^e^ | 0.019 |
|  | Unemployed or reduced working hours | 152 (20.8) | 1.84 (1.45-2.35)^e^ | 1.04 (0.71-1.51)^e^ | 0.843 |
|  | Other ^c^ | 151 (19.1) | 1.84 (1.45-2.33)^e^ | 1.55 (1.06-2.25)^e^ | 0.023 |

^a^ Not including individuals with missing data on any variables in model 2 (the fully adjusted model), to allow for direct comparison.

^b^ Adjusted for country, age in 4 levels, whether the participant experienced the relevant IPV outcome in the three months before COVID-19, employment status before COVID-19 and cannabis use frequency before COVID-19 measures.

^c^ Includes those who changed job, were paid but unable to work, or answered “other”. Those selecting “other” were unable to specify further.

^d^ N=2973

^e^ N=5112

**M** **Associations between outcome nonresponse and other variables**

**Table M:** Association between each variable of interest and non-response on the outcome variable (IPV experience during COVID-19).

| **Characteristics** | **Total number missing outcome data (%)** | **Number of cis-women who are in a relationship and missing outcome data (%)** |
| --- | --- | --- |
| **Age (years)** |  |  |
| 18-24 | 1459 (43.8) | 262 (13.4) |
| 25-30 | 1022 (30.4) | 235 (9.6) |
| 31-40 | 883 (25.5) | 251 (9.2) |
| >40 | 978 (31.5) | 269 (11.7) |
| **Sexual orientation** |  |  |
| Heterosexual | 3154 (30.4) | 734 (9.7) |
| Other sexual orientation ^a, b^ | 1097 (43.0) | 251 (15.8) |
| **Ethnicity** |  |  |
| Majority in country | 405 (38.7) | 523 (9.5) |
| Minority in country | 2377 (30.7) | 111 (15.6) |
| Unclear | 353 (36.2) | 112 (15.6) |
| **Educational attainment** |  |  |
| Primary or less than primary | 221 (30.7) | 51 (10.1) |
| Secondary (partial or completed) | 930 (36.6) | 205 (11.9) |
| University or college (partial or completed) | 3079 (32.1) | 735 (10.7) |
| Other ^a^ | 171 (31.1) | 49 (12.0) |
| **Employment status before the pandemic** |  |  |
| Employed or self-employed | 2444 (28.8) | 635 (9.9) |
| Unemployed or informal work | 1033(43.1) | 91 (13.4) |
| Student | 371 (37.0) | 173 (12.2) |
| Other or multiple ^a^ | 564 (37.2) | 143 (13.7) |
| **Change in employment status during the pandemic** |  |  |
| Continued in same work and site ^c^ | 1282 (29.1) | 328 (10.0) |
| Working from home (completely or partially) | 1457 (32.3) | 342 (10.6) |
| Lost employment or working reduced hours | 734 (37.9) | 185 (14.0) |
| Other ^a, d^ | 895 (36.4) | 181 (11.0) |
| **Transactional sex before the pandemic** |  |  |
| Never | 3691 (30.4) | 862 (9.7) |
| Yes | 105 (45.1) | 42 (29.0) |
| **Marital status** |  |  |
| Not married | 4037 (43.3) | 682 (12.5) |
| Married | 298 (7.6) | 298 (7.6)^f^ |
| **Children** |  |  |
| None | 3454 (38.7) | 589 (11.0) |
| Any | 1159 (23.2) | 453 (10.8) |

**Continued…**

| **Characteristics** | **Number missing outcome data (%)** | **Number of cis-women who are in a relationship and missing outcome data (%)** |
| --- | --- | --- |
| **Living with partner during the pandemic** |  |  |
| Living with partner | 283 (5.0) | 598 (17.2) |
| Not living with partner | 3554 (52.0) | 283 (5.0) |
| Unclear | 560 (60.0) | 881 (9.7) |
| **Household composition during the pandemic** |  |  |
| Unchanged | 3569 32.4) | 818 (10.4) |
| Different to before the pandemic | 820 (36.5) | 209 (13.6) |
| **Perceived household income before the pandemic** |  |  |
| Less than average | 1468 (36.3) | 345 (12.6) |
| Average | 1392 (31.7) | 286 (9.2) |
| Higher than average | 840 (26.2) | 191 (7.8) |
| **Frequency drinking alcohol before the pandemic** |  |  |
| Never | 1053 (35.4) | 268 (12.7) |
| Up to 4 times a month | 2616 (32.0) | 601 (10.3) |
| Multiple times a week | 729 (32.5) | 170 (10.6) |
| **Cannabis use before the pandemic** |  |  |
| Never | 3481 (31.4) | 820 (10.2) |
| Up to 4 times a month | 671 (40.8) | 142 (13.8) |
| Multiple times a week | 169 (36.0) | 49 (15.4) |
| **IPV experience in the three months before the pandemic** |  |  |
| Experienced IPV | 363 (18.5) | 165 (2.4) |
| Did not experience IPV | 590 (7.9) | 107 (6.6) |
| **Average COVID-19 response stringency experienced prior to completing the survey ^e^** |  |  |
| Low | 1618 (37.8) | 407 (13.7) |
| Medium | 1158 (25.1) | 223 (6.5) |
| High | 1636 (36.2) | 412 (13.1) |
| **Time spent with social distancing measures in place prior to completing the survey** |  |  |
| 1-6 months | 854 (37.6) | 201 (12.7) |
| 7-9 months | 2774 (31.8) | 687 (10.9) |
| 10-12 months | 784 (32.3) | 154 (9.3) |
| **Total** | 4412 (32.9) | 1042 (10.9) |

**Table M continued:**

^a^ Participants selecting “other” for any item were unable to specify their situation.

^b^ Includes participants identifying as lesbian, gay, bisexual, questioning or unsure, asexual, pansexual or other.

^c^ Including those who were retired before COVID-19 social distancing measures.

^d^ Includes participants who have changed job, are employed but unable to work, or who selected other.

^e^ Calculated using a summary of the number and intensity of social distancing measures and containment policies experienced by the participant over the period of COVID-19 social distancing.

^f^ Includes married and living together, married not living together, married and separated, not married and living together, relationship not married or living together, other or multiple. Excludes women identifying as single, widowed or divorced.

**N Sensitivity of conclusions to sampling method**

**Table N**: Association between changes in employment status and experiencing intimate partner violence during COVID-19 social distancing measures among cis-gender women who participated in the I-SHARE survey 2020-21, organised according to the sampling method used for their recruitment.

| **Sampling method** | **Change in employment status** | **Individuals experiencing IPV (%)** | **OR adjusted for country**  **(95% CI)^a^** | **Fully adjusted OR**  **(95% CI)^b^** | **Wald test p-value** |
| --- | --- | --- | --- | --- | --- |
| All | Continued in same work and site/ retired | 376 (12.0) | 1 | 1 |  |
|  | Working (completely or partially) from home | 482 (15.8) | 1.24 (1.06-1.44)^d^ | 1.40 (1.12-1.74)^d^ | 0.003 |
|  | Unemployed or reduced working hours | 250 (20.8) | 1.66 (1.37-2.00)^d^ | 1.15 (0.87-1.53)^d^ | 0.313 |
|  | Other^c^ | 274 (17.5) | 1.47 (1.22-1.76)^d^ | 1.46 (1.11-1.94)^d^ | 0.007 |
| Convenience | Continued in same work and site/ retired | 296 (12.9) | 1 | 1 |  |
|  | Working (completely or partially) from home | 387 (15.2) | 1.09 (0.92-1.30)^e^ | 1.25 (0.98-1.60)^e^ | 0.077 |
|  | Unemployed or reduced working hours | 189 (19.8) | 1.49 (1.20-1.84)^e^ | 1.13 (0.83-1.55)^e^ | 0.437 |
|  | Other^c^ | 219 (17.2) | 1.34 (1.09-1.65)^e^ | 1.39 (1.01-1.90)^e^ | 0.044 |
| Population representative/ online panel | Continued in same work and site/ retired | 80 (9.6) | 1 | 1 |  |
|  | Working (completely or partially) from home | 95 (18.3) | 2.00 (1.40-2.85)^f^ | 2.13 (1.27-3.57)^f^ | 0.004 |
|  | Unemployed or reduced working hours | 61 (24.2) | 2.44 (1.61-3.71)^f^ | 1.14 (0.61-2.10)^f^ | 0.684 |
|  | Other^c^ | 55 (18.8) | 1.99 (1.32-2.99)^f^ | 1.57 (0.86-2.88)^f^ | 0.142 |

^a^ Not including individuals with missing data on any of the variables in the fully adjusted model for this variable, to allow for direct comparison.

^b^ Adjusted for country, age in 4 levels, whether the participant experienced IPV in the three months before COVID, employment status before COVID-19 measures, and cannabis use frequency before COVID-19 measures.

^c^ Includes those who those who changed job, were paid but unable to work or answered “other”. Those who selected “other” were unable to specify further.

^d^ N= 8362 from 25 countries.

^e^ N=6607 from 20 countries.

^f^ N=1755 from 6 countries.

**O Sensitivity of conclusions to missing data definition**

**Table O**: Association between changes in employment status and experiencing intimate partner violence during COVID-19 social distancing measures among cis-gender women who participated in the I-SHARE survey 2020-21, organised according to different approaches to outcome missing data inclusion.

| **Missing data definition** | **Change in employment status** | **Individuals experiencing IPV (%)** | **OR adjusted for country**  **(95% CI)^a^** | **Fully adjusted OR**  **(95% CI)^b^** | **Wald test p-value** |
| --- | --- | --- | --- | --- | --- |
| Exclusion approach in the main analysis: Only participants with complete IPV responses included in the analysis. | Continued in same work and site/ retired | 376 (12.0) | 1 | 1 |  |
|  | Working (completely or partially) from home | 482 (15.8) | 1.24 (1.06-1.44)^d^ | 1.40 (1.12-1.74)^d^ | 0.003 |
|  | Unemployed or reduced working hours | 250 (20.8) | 1.66 (1.37-2.00)^d^ | 1.15 (0.87-1.53)^d^ | 0.313 |
|  | Other ^c^ | 274 (17.5) | 1.47 (1.22-1.76)^d^ | 1.46 (1.11-1.94)^d^ | 0.007 |
| Inclusion approach sensitivity test: Participants who answer ‘yes’ to any IPV indicator are included as a case. Participants who answer ‘no’ to some items, and are missing data on others, are included as controls provided they have not answered yes to any item. | Continued in same work and site/ retired | 387 (12.1) | 1 | 1 |  |
|  | Working (completely or partially) from home | 493 (15.7) | 1.20 (1.03-1.39)^e^ | 1.35 (1.09-1.67)^e^ | 0.006 |
|  | Unemployed or reduced working hours | 257 (20.8) | 1.61 (1.34-1.94)^e^ | 1.20 (0.91-1.57)^e^ | 0.192 |
|  | Other ^c^ | 282 (17.7) | 1.44 (1.21-1.73)^e^ | 1.49 (1.13-1.95)^e^ | 0.004 |

^a^ Not including individuals with missing data on any of the variables in the fully adjusted model for this variable, to allow for direct comparison.

^b^ Adjusted for country, age in 4 levels, whether the participant experienced IPV in the three months before COVID, employment status before COVID-19 measures, and cannabis use frequency before COVID-19 measures.

^c^ Includes those who those who changed job, were paid but unable to work or answered “other”. Those who selected “other” were unable to specify further.

^d^ N= 8362 from 25 countries.

^e^ N=8664 from 25 countries.
